# Phase change and flowering in woody plants of the New Zealand flora

**DOI:** 10.1093/jxb/erv472

**Published:** 2015-10-28

**Authors:** Paula E Jameson, John Clemens

**Affiliations:** 1 School of Biological Sciences, University of Canterbury, Christchurch, New Zealand; 2 Christchurch Botanic Gardens, Christchurch City Council, Christchurch, New Zealand; 3 Swedish University of Agricultural Sciences

**Keywords:** ABC model, *Clianthus*, *Eucalyptus*, flowering, gibberellin, growth habit, heteroblasty, homoblasty, *Metrosideros*, microRNA, *Pachycladon*, phase change, *Phormium*, photoperiod, reproduction, *Sophora*

## Abstract

Heteroblastic and homoblastic woody plants from the New Zealand flora provide a rich playground for testing hypotheses relating to phase change and flowering.

## A brief introduction to the New Zealand flora

Over a decade ago we were invited by Georges Bernier to provide a ‘minireview of research activity’, which we presented as ‘A woody perennial perspective of flowering’ (Clemens *et al.*, 2002). That review focused on *Metrosideros excelsa.* Here, we cover some of the research carried out subsequently on this and other species, and look towards the future use of some of the peculiarities of the New Zealand flora in extending our knowledge of phase change.

The New Zealand flora, which has relatively low numbers of families and genera for the area of the country, has often been described as ‘depauperate’ and predominated by small white or yellow flowers together with a paucity of butterflies and long-tongued bees (Webb and Kelly, 1993; Lee *et al.*, 2001; Craine *et al.*, 2006). However, there are several tree genera with brightly coloured flowers that are bird pollinated (Kelly *et al.*, 2010). These include *Metrosideros* (Myrtaceae), *Sophora* (Fabaceae), and *Clianthus* (Fabaceae).

The red-flowering *M*. *excelsa* (pōhutukawa) and *Clianthus* (two species of kakabeak), and the yellow-flowering *Sophora* (several species of kowhai) hold iconic status for both indigenous Māori of New Zealand and for the population as a whole. The pōhutukawa is popularly known as the New Zealand Christmas tree as it flowers about Christmas time in the Southern Hemisphere summer. A lone pōhutukawa holds particular significance to Māori (Simpson, 1994). The tree, reputed to be 800 years old grows at Cape Reinga, the northernmost tip of the North Island. Te Reinga translates as the ‘leaping place of spirits’. The legend is that, on death, the spirit of the person travels to this most northern point, descends by way of the roots of this tree to the underworld, and then travels to the homeland of Hawaiiki-a-nui.

The website maoriplantuse.landcareresearch.co.nz provides interesting details about these plants. All three had significant use in pre-European times with the red flowers of pōhutukawa and kakabeak used for ornamentation. Both pōhutukawa and kowhai were used medicinally and for wood, although the kowhai seeds and wood are toxic. The yellow flowers of the kowhai were used as a dye as well as to signal the time to plant the staple root crop kūmara (sweet potato, *Ipomoea batatas*). Kakabeak may have been cultivated for food and it was widely grown in both pre- and post-European settlements (Colenso, 1885). Several kakabeak cultivars are grown as garden plants throughout New Zealand, for example, the red-flowered ‘Kaka King’, ‘White Heron’, and the pink ‘Flamingo’.

Unfortunately, all three genera now hold species that are nationally threatened (de Lange *et al.*, 2013). Only 153 plants of kakabeak were detected in the wild in 2005 (Song *et al.*, 2008b) growing in relatively inaccessible regions of the East Coast of the North Island. Indeed, in 1885, Colenso wrote that he had never seen *Clianthus* growing ‘truly wild and common’. Kakabeak is classified as critically threatened nationally and its continued survival in the wild is described as conservation-dependent (de Lange *et al.*, 2013).

Banks and Solander were the first to collect kakabeak in 1769, but *Clianthus puniceus* was not described until 1835. Colenso grew kakabeak from locally cultivated plants in Northland and then later further south in Hawkes Bay. He contrasted the more northern form with the more southern form and described the latter as *C. maximus* (Colenso, 1885). In 1899, Kirk reduced *C. maximus* to a variety of *C. puniceus.* Subsequently, Heenan (2000) re-instated the two species, using *C. puniceus* to describe the one specimen from Kaipara Harbour (Northland), and *C. maximus* to describe the East Coast, including Hawkes Bay, populations. However, our data, based on the presence/absence of a seven-base-pair deletion in intron 2 of *LEAFY*, indicate that the Kaipara Harbour *Clianthus* is not a species distinct from the extant populations found in the wild in New Zealand. We suggested that it is more likely to be a translocation, and that there is a morphological gradation from north to south of a single species of *Clianthus* (Song *et al.*, 2008b).

The kowhai is often referred to as New Zealand’s unofficial national flower. Several species are regarded as naturally uncommon (de Lange *et al.*, 2013) but, like kakabeak, it is widely grown as a garden plant. By contrast, the pōhutukawa, unlike some other species of *Metrosideros*, is not formally regarded as threatened (de Lange *et al.*, 2013), although its range has been greatly reduced owing to clearance and damage from introduced herbivores (Bylsma *et al.*, 2014). The pōhutukawa has been widely planted as a road and park tree outside its natural range.

We were interested in the potential of these plants as cultivated species for cut flower or flowering plant production, but little work had been carried out relating to phase change, floral induction or flower development. A comparative analysis is offered in this commentary to contrast the differences in the timing of and cues for floral induction, and in floral initiation and floral organ differentiation. However, we show that, despite these contrasting behaviours, gene expression during flower development in all three species adhered to predictions based on the ABC model of flower development. The data provided are for plants studied in Palmerston North, New Zealand (40° 23' 6'' S, 175° 36' 51'' E). The comparative analysis of flowering in the three woody genera was first drawn together for a talk entitled ‘The ABCs of flowering in three iconic New Zealand species’ and was presented by PEJ as the 35th John Smaillie Tennant Lecture on the occasion of the 90th Birthday of the Department of Botany, University of Otago, New Zealand, 11 September 2014. However, in this commentary, we also introduce research on a wider range of species, and particularly on phase change.

## Morphological analysis

**Fig. 1.**
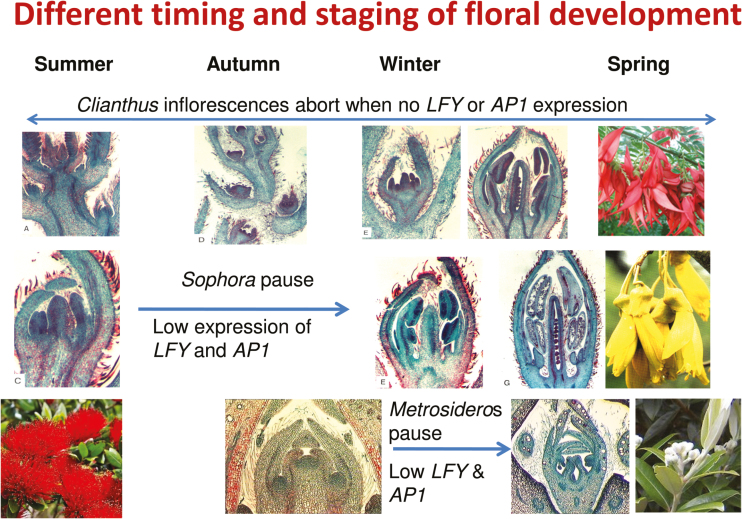
Temporal pattern of floral development of *Clianthus, Sophora*, and *Metrosideros* over an annual cycle. Floral initiation in *Sophora* and *Clianthus* occurred soon after the previous season’s flowering in spring whereas that in *Metrosideros* was in autumn. The relationship of the stage of development to reduced expression of *LEAFY-* and *APETALA1-*equivalents is shown.

Over an annual cycle it was clear that floral initiation by the kowhai and kakabeak (referred to below as *Sophora* and *Clianthus*, respectively) occurred soon after the previous season’s flowering in spring, but that of pōhutukawa (referred henceforth as *Metrosideros*) was several months later in autumn (Fig. 1). In *Sophora*, floral primordia were evident in spring (October), and floral organ initiation occurred rapidly and continued through to mid-summer (end of January). Development then paused through the autumn and early winter, but in mid-winter (July) rapid organogenesis occurred although the petals remained small until a period of rapid elongation just prior to flowering in early spring (late August/early September) (Song *et al.*, 2008a).

While *Clianthus* initiated inflorescences continuously, and within these floral primordia were evident, organogenesis did not occur until late autumn/early winter. Organogenesis continued through to flowering in early spring (September) and, as with *Sophora*, petals were slow to elongate (Song *et al.*, 2011). In contrast to both *Sophora* and *Clianthus*, cymule primordia were not detected in *Metrosideros* until the autumn, and then further organ initiation and differentiation was delayed until late spring (Sreekantan *et al.*, 2001).

## Environmental responsiveness

Experimentation showed that *Metrosideros* is a facultative short-day plant (Henriod *et al.*, 2000), with flower numbers being influenced by cool (mean 15 °C), not cold (mean 10 °C) temperatures and irradiance, as well as by bud size (Henriod *et al.*, 2003). While *Clianthus* inflorescences were continuously produced, most aborted and only those produced during a few weeks in autumn continued to elongate and commence organ initiation (Song *et al.*, 2011). Floral initiation was, therefore, not a short-day response but it is likely that the subsequent organ initiation was a response to the cooler temperatures of late autumn. Similarly, floral initiation in *Sophora* was not a response to short-day signals, but flower development required cooler conditions: floral initiation and organ differentiation occurred in spring but organogenesis in *Sophora* was delayed until mid-winter (Song *et al.*, 2008a).

Lee *et al.* (2001) suggest that there is a relative lack of cold tolerance in most of the modern New Zealand woody flora, which is of warm-temperate/subtropical affinity. They suggest that much of New Zealand’s floral richness was lost as the climate cooled during the Late Miocene-Pliocene (Lee *et al.*, 2001), so it is interesting to identify a requirement for cooler temperatures for full floral development and the consequent deferment of flowering until early spring. However, this is not a vernalization requirement, as floral initiation started in all three species prior to winter chilling.

## The ABC model

Within the three New Zealand species, the extended period of floral development, the different timing of organ initiation and development, and the ‘pause’ in development during the winter months provided an opportunity to assess the expression of *LEAFY*, and the relevant equivalents of the A (*APETALA1*), B (*PISTILLATA*), and C (*AGAMOUS*) genes associated with floral morphogenesis. We showed that the A-, B-, and C-equivalents in *Sophora* and *Clianthus* were expressed in sepals, petals, stamens, and carpels (Song *et al*., 2008a, 2011) as predicted by the floral organ identity model (Bowman *et al.*, 1991; Coen and Meyerowitz, 1991).

In *Arabidopsis* all organs within a whorl develop simultaneously in the order of sepal, petal, stamen, and carpel. By contrast, in both *Sophora* and *Clianthus* this is not the case. Following sepal and petal initiation, only the outer stamen primordia initiate, followed then by the carpel, and then the inner stamens; in addition, the carpel primordium enlarges rapidly whereas petal enlargement is significantly delayed (Song *et al.*, 2008a, 2011). One might then predict more ‘C’ gene expression before that of ‘B’. A close inspection of the expression profiles shows that the expression of *StAG* and *CmAG* increases more rapidly than that of *StPI* and *CmPI*, respectively, tempting the suggestion of a causative relationship between early carpel primordial enlargement and precocious expression of the ‘C’ gene (Song *et al.*, 2008a, 2011).

**Fig. 2.**
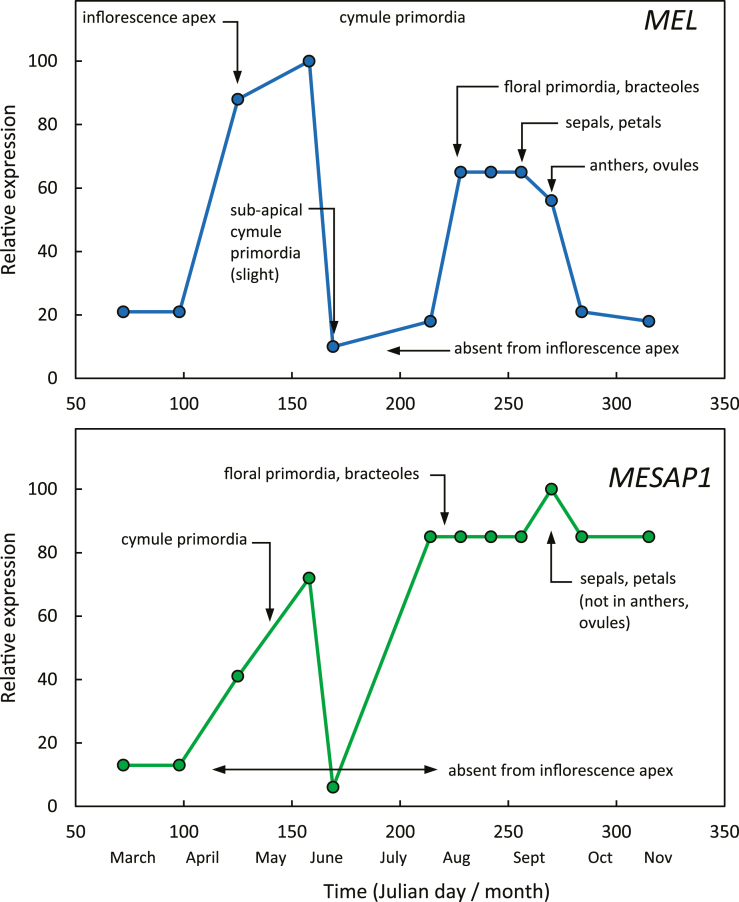
Expression of *Metrosideros excelsa LEAFY* (*MEL*) and *APETALA1* (*MESAP1*) over time and space during floral development. Data compiled from Sreekantan (2002).

Combining the *in situ* hybridization, northern, RT-qPCR, and morphological data for *Metrosideros* (Sreekantan *et al.*, 2004; Jaya *et al.*, 2011), both *MeLEAFY* (*MEL*) and *MeAP1* (*MESAP1*) were expressed as the cymule primordia developed during the autumn, were not expressed during much of the winter period, but increased in expression towards the end of winter. *MESAP1* was expressed in sepals and petals but not anthers or ovules, as might be expected of an ‘A’ class gene (Fig. 2).

In *Sophora*, where floral organ initiation occurred throughout the summer, both *StLFY* and *StAP1* were expressed, but expression had declined by the end of summer by which time floral development had synchronized but then paused. Floral organ differentiation coincided with the increase in expression of *StLFY*, *StAP1*, *StAG*, and *StPI* (Song *et al.*, 2008a). In *Clianthus*, low level *CmLFY* expression occurred while inflorescences were proliferating, but floral development coincided with marked increases in *CmLFY*, *CmAP1*, *CmAG*, and *CmPI* (Song *et al.*, 2011).

In all three species, floral organ initiation required the expression of both *LFY* and *AP1* (Fig. 1). The ‘pause’ in flower development in *Sophora* and *Metrosideros* occurred at a different developmental stage (following floral organ initiation and cymule primordia initiation, respectively), and coincided with low expression of both *LFY* and *AP1*. Subsequent floral development in all three species required the expression of *LFY* and the ‘A’, ‘B’, and ‘C’ genes, respectively. This confirmed the dual role initially suggested by Bowman *et al.* (1993) for *AP1* in floral meristem specification and floral organ development and expanded by Kaufmann *et al.* (2010) for *AP1*, suggesting that its actions are initially that of a repressor of floral repressors but then as a subsequent activator of regulatory genes involved in floral organ formation. While Mizzotti *et al.* (2014) question the universality of class A-function genes beyond *Arabidopsis*, *AP1* in our three woody species conforms to such a gene, although functional analysis has not been carried out due to our respecting the three species as tāonga (‘treasures’) to Māori, and so not transforming the *AP1* gene into *Arabidopsis* for functional analysis.

## The gibberellin pathway

In *Arabidopsis*, gibberellin is required for flowering under non-inductive short days (Blázquez *et al.*, 1998). We have shown that gibberellin has a promotive effect on flowering in two monocots: *Chionochloa macra* (Martin *et al.*, 1993) and *Phormium cookerianum* (the endemic New Zealand mountain flax; Harris *et al.*, 2009). In mountain flax, floral induction was unaffected by either temperature or daylength – size being the determinant of competence to flower. However, GA_3_ promoted flowering of small and medium-sized fans that would otherwise not have flowered. Expression of *LFY* correlated positively with size of fan and ability to flower and the application of GA_3_ accelerated the increase in *LFY* (Harris *et al.*, 2009). However, the final level of *LFY* did not correlate with the proportion that flowered, a pattern similar to *LtLFY* in *Lolium temulentum* (Gocal *et al.*, 2001) and *RFL* in rice (Kyozuka *et al.*, 1998).

In contrast to the monocots, we have shown that the application of gibberellins caused floral buds of *Metrosideros* to abort (Clemens *et al.*, 1995), a similar situation subsequently shown in other woody dicots, particularly fruit trees (Goldberg-Moeller *et al.*, 2013, and references therein). Gibberellin caused an increase in *LFY* expression in non-flowering juveniles but not of *AP1* (Sreekanatan *et al.*, 2004). Our analysis of all three flowering woody species indicates that flowering will only progress if both *LFY* and *AP1* are expressed. Interestingly, GA_1_ was not detectable in individual buds of reproductively competent plants in July, during the mid-winter ‘pause’ in floral development in *Metrosideros*, but was at its highest level during spring in actively growing vegetative shoots and in flower buds when *LFY* expression had again increased (Sreekantan *et al.*, 2004). In their dual model for GA activity during *Arabidopsis* flowering, Yamaguchi *et al.* (2014) suggest that *LFY* is initially up-regulated by GA, but *LFY* subsequently directs the destruction of GA, thus allowing the accumulation of DELLA proteins required for the subsequent up-regulation of *AP1* and for flowering to progress (see Fig. 4F in Yamaguchi *et al.*, 2014). While the application of GA did not promote the juvenile to adult vegetative transition in *Metrosideros* (Clemens *et al.*, 1995), it did inhibit the progression of floral buds to flowers, which would indicate that an over-supply of GA is inhibitory for the progression of floral development, as suggested by Yamaguchi *et al.* (2014). To determine where the GA_1_ that we detected is acting in individual flower buds harvested 2–3 weeks before anthesis, careful dissection and analysis of the floral buds will be required. However, at this stage of development, all floral organs had differentiated and were rapidly expanding, and the gibberellin may be involved in anther development (Sakata *et al.*, 2014, and references therein).

## Phase change

While the long juvenile phase exhibited in woody species is a serious constraint to plant breeding and to the production of floricultural crop plants raised from seed, it offers opportunities to characterize and elucidate the control of phenotypic transitions occurring in leaf and habit in the vegetative plant (vegetative phase change) alongside, and not necessarily coupled with, reproductive transition. As observed by Zotz *et al.* (2011), most plant species show a relatively subtle and gradual change in character between juvenile and adult vegetative states. However, there is a significant number of species in which there is a more-or-less abrupt demarcation between the two states (Zotz *et al.*, 2011). Goebel (1900) described such species as ‘heteroblastic’, and those with an abrupt change in habit were described by Philipson (1964) as exhibiting ‘habit heteroblasty’. However, in the more recent literature the term ‘heteroblasty’ has come to be used for even the most gradual of morphological changes (e.g. Poethig, 2013) where the term ‘homoblastic’ (*sensu stricto*Goebel, 1900) should be used. Zotz *et al.* (2011) suggest the terminology used by Goebel (1900) should be retained and demonstrate schematically the abrupt or gradual changes taking place against a size/age profile (see Fig. 2 in Zotz *et al.*, 2011). According to Cockayne (1911), ‘About two hundred species of New Zealand vascular plants, belonging to thirty-seven families, show a more or less well-marked distinction between the juvenile and adult stages of development, while in perhaps one hundred species the differences are very great indeed’. As New Zealand plant biologists, where the term heteroblasty describes a significant aspect of our flora, we fully support the use of Goebel’s original terminology (Horrell *et al.*, 1990b; Darrow *et al.*, 2002; Jaya *et al.*, 2010b; Sooda *et al.*, 2011; see also Appendix 1 in Zotz *et al.*, 2011).

The confusion in the literature highlights the complexity of the situation, but now that a marker of the vegetative phase has been identified (Wu *et al.*, 2009), some of these anomalies may be tested. In the annual model species (*Arabidopsis thaliana* and *Zea mays*), microRNA156 (miR156) is considered to be a master regulator of vegetative phase change (Poethig, 2013). MicroRNAs are short, single-stranded RNAs that regulate target gene expression at the post-transcriptional level. MicroRNA156 and miR172 have distinctive roles in maintaining the juvenile vegetative phase and initiating reproduction, respectively (Zhu and Helliwell, 2011; Spanudakis and Jackson, 2014). Transgenic experiments clearly show that high levels of miR156 maintain the juvenile vegetative state and delay flowering, whereas high levels of miR172 promote flowering and vice versa: plants with reduced miR156 flower earlier; those with reduced miR172 flower later (Yu *et al.*, 2015, and references therein). While models show that a balance between miR156 and miR172 controls the timing of the juvenile to adult vegetative phase change (Zhu and Helliwell, 2011; Teotia and Tang, 2015), there appears to be little experimental work confirming this within the same plant (Wu *et al.*, 2009) or in woody perennial species (Wang *et al.*, 2011). As we have shown with the ABC genes, New Zealand native plants should also be ideal subjects to test the utility of microRNAs and their downstream regulated genes as markers of the different states, as well as contributing information relating to the juvenile to adult vegetative phase transition and the transition from non-reproductive to reproductive.

In addition to heteroblasty, the New Zealand flora has an unusually high proportion of species that exhibit a pronounced divaricating habit in the juvenile (Wardle, 1991; Goldberg *et al.*, 2008). Divaricate plants typically have a wide branching angle, closely interlaced, springy, branches, and small leaves concentrated in the interior of the plant (Kelly, 1994). Tree species exhibiting the divaricating habit usually convert to an arborescent habit (tree) prior to flowering. However, some species appear to have ‘lost’ the ‘adult’ tree form (Cockayne, 1911) and yet are capable of flowering.

For example, different species of *Sophora* exhibit different life strategies: *S. prostrata* Buchan, *S. microphylla* Ait., and *S. tetraptera* J. Mill differ markedly from each other in their juvenile forms (Cockayne, 1911; Godley and Smith, 1978). *Sophora prostrata* grows as a prostrate, divaricating shrub throughout its ontogeny whereas *S. tetraptera* does not have a divaricating juvenile vegetative phase and forms a small tree with spreading branches about 12 m in height (Allan, 1961). *Sophora microphylla* on the other hand is variable in form depending on location, and may or may not have a juvenile divaricating form. However, the adult is a tree up to 10 m in height (Salmon, 1980; Carswell *et al.*, 1996). Such inter- and intra-specific variation provides opportunities to test the miR156/miR172 model beyond the classical perennial phase change models (Wang *et al.*, 2011). Moreover, and similar to a response in ivy (Rogler and Hackett, 1975; Horrell *et al.*, 1990a), the application of gibberellin can induce a change from the adult form and foliage of several heteroblastic species to that more indicative of the divaricating juvenile (Horrell *et al.*, 1990b), providing further material for microRNA analysis.

We also have the opportunity to use species in which experimental manipulations have been shown to affect both the vegetative to adult transition and also the transition to flowering. In contrast to heteroblastic species, *Metrosideros excelsa* exhibits homoblastic behaviour in the strict sense of Goebel (1900), since vegetative phase change occurs gradually between juvenile and adult states (Clemens *et al.*, 1999). Experimentally, vegetative phase change can be hastened by growing plants with a single stem (Clemens *et al.*, 1999; Kubien *et al.*, 2007; Jaya *et al.*, 2011), whereas flowering can be advanced by subsequently allowing such plants to branch (Jaya *et al.*, 2011). In contrast to manipulating phase change by transgenic means (Lauter *et al.*, 2005; Wu and Poethig, 2006; Wu *et al.*, 2009; Wang *et al.*, 2011), an ability experimentally to manipulate the transition of the juvenile to adult phase change, as well as the timing of reproduction, provides useful material to elaborate on the role of both miR156 in maintaining the juvenile phase and miR172 in the transition to the adult phase and to reproduction. Moreover, *Metrosideros* also exhibits ‘rejuvenation’ when elite selections are micropropagated from reproductive plants (Oliphant *et al.*, 1991; Clemens *et al.*, 1995). Such micropropagated plants are slow to return to flowering. In this case, the presence of miR156, or perhaps a change in the ratio between miR156 and miR172 might confirm whether this is a complete rejuvenation as both truly juvenile and truly adult and reproductive material are available for comparison.

In contrast to *Metrosideros*, experimentally single-stemmed plants of another member of the Myrtaceae, *Eucalyptus occidentalis*, do not undergo the transition from juvenile to adult vegetative foliage (Jaya *et al.*, 2010a, b, 2011). Moreover, the plants bearing juvenile foliage flowered just as rapidly as branched plants with adult foliage. The floral transition in these plants is clearly independent of vegetative phase change. *Eucalyptus occidentalis* provides a unique opportunity to assess the independence of vegetative phase transitions and the transition from vegetative to reproductive, utilizing miR156 and miR172 as markers. The model proposed by Wang (2014) shows the potential for maintenance of juvenility within the leaf and transition to reproduction in the shoot apical meristem based on the different targets of downstream factors (see Fig. 2 in Wang, 2014).

Species that are distinctly heteroblastic (Goebel, 1900*sensu stricto*; Zotz *et al.*, 2011) such as *Elaeocarpus hookerianus* Raoul (pokaka), but which exhibit a third ‘adolescent’ state (Day *et al.*, 1997), may well be ideal plants to confirm the models that show both miR156 and miR172 expressing as plants transition from the juvenile vegetative to the adult vegetative states. Are both miR156/miR172 present in the non-reproductive adult vegetative state and miR172 expressing alone in the reproductive state (Teotia and Tang, 2015)?

Interestingly, analyses of cytokinins in leaves from juvenile and mature forms of *E. hookerianus* indicate that the divaricating juvenile form contained more active forms and fewer storage forms of cytokinin than did leaves from the transitional and adult arborescent forms (Day *et al.*, 1995). Further, in *Sophora***,** the divaricate form showed more active forms relative to storage forms of cytokinin (Carswell *et al.*, 1996). We highlight this because, recently, Zhang *et al.* (2015) suggested that, as leaves age, the decline in shoot regenerative capacity in tissue culture is related to the decrease in miR156 and the up-regulation of a previously repressed gene that directly impacts on the cytokinin signalling pathway, thereby reducing plant responsiveness to cytokinin in culture in the older leaves (Zhang *et al.*, 2015). It will be of interest to determine if similar correlations exist between cytokinin forms and/or sensitivity and miR156 in the New Zealand native species.

Another endangered species (Molloy *et al.*, 1999; Luo *et al.*, 2003), *Pachycladon exile* (Heenan) Heenan & A.D. Mitch. (Family Brassicaceae), provides a perennial herb related to *Arabidopsis lyrata*, which concurrently has primary and secondary axes that are floral, but tertiary axes that remain vegetative, green, and perennating (Sooda *et al.*, 2011). Are such perennating structures vegetatively juvenile (high miR156?) and not competent to respond to flowering signals, or adult (high miR172) but somehow repressed, or carrying an intermediate level of both miR156 and miR172, i.e. vegetatively adult but not competent to respond reproductively?

## Conclusions

The New Zealand flora provided flowers with quite different temporal patterns of development on to which could be overlaid the ABC model of floral development. Similarly, the flora offers species with which to assess the definitive nature of miR156 and miR172 and their downstream target genes in driving vegetative phase change in heteroblastic and homoblastic species, and their floral transition.

Work on these woody perennials emblematic of New Zealand was triggered by a call from industry for their greater global floricultural prominence. Work on woody perennials is famously dogged by slow maturation, long developmental cycles once a reproductive state is reached, and branch superstructures that might not lend themselves to commercial development. Paradoxically, these are the very phenomena that allowed us to examine and separate the morphological and genetic information we sought. Nonetheless, the combination of laboratory and horticultural skills, along with the intellectual endeavour necessary to obtain our results, are a testament to the dedication of students and co-workers who worked in a cultural and regulatory environment where genetic modification of these native tāonga could not be countenanced. We would like to dedicate this work to two colleagues who, in different ways, supported this work, Dr Garry Burge and Professor Michael McManus, both of whom have passed away.
